# Collectanea Medica, Consisting of Anecdotes, Facts, Extracts, Illustrations, Queries, Suggestions, &c.

**Published:** 1817-05

**Authors:** 


### COLLECTANEA MEDICA,

CONSISTING OF

### ANECDOTES, FACTS, EXTRACTS, ILLUSTRATIONS, QUERIES, SUGGESTIONS, &c.

RELATING TO THE

***History or the Art of Medicine, and the Auxiliary Sciences.***

---

### *Biographical Memoirs of the Right Reverend Dr. Watson, Bishop of Llandaff.*

IT is not a little remarkable that three men to whom we are indebted for some of the earliest improvements in modern chemistry, should have suffered by their supposed attachment to political inquiries. The reader will at once recollect Lavoisier, Priestley, and Watson. Of the first we can only say, *Hinc illæ lachrymæ!* The second was certainly exposed to persecution; but we must admit not more than his sacred calling should have taught him to endure. However he was the only sufferer by this impatience, which induced him to abandon his country, but never to retort the insults and calumnies which he received from others. The third, whose memoirs we are preparing to offer, submitted, like a true divine, to retain the lowest seat in the episcopal bench, from which he was never translated.

"Richard Watson was born in August, 1737, at Heversham, in Westmorland, five miles from Kendal, in which town his father, a clergyman, was master of the Free Grammar School. His father superintended the whole of his early education till the year 1754, when he went to Trinity College, Cambridge. Here he distinguished himself by close application to study, residing constantly till made a scholar in May, 1757. He became engaged with private pupils in November following; and took the degree of B. A. in January, 1759. On this occasion he held a distinguished place, being Second Wrangler. He was elected Fellow of Trinity College in October, 1760; and was appointed assistant tutor to Mr. Backhouse in November of the same year. He took the degree of M.A. in 1762; and was made Moderator for the first time in October following.

"In the year 1764, he became a candidate for the Professorship of Chemistry. I have been told that the late Dr. Paley, who afterwards distinguished himself so much by his writings in the departments of moral philosophy and theology, was a candidate for the same chair. Neither of these eminent men had paid any previous attention to the study of chemistry. Dr. Paley boasted at the time that he was better acquainted with it than Dr. Watson; for he could perform one chemical process at least, since he knew how to make *red ink;* while his antagonist, he believed, did not know so much. Dr. Watson, however, carried the election; and began the study of practical chemistry with so much assiduity, that he very materially injured his health. I have been frequently amused with the history of his first chemical campaign. He could not succeed in his earliest attempts at experimenting. His retorts broke, his liquids were spilled, his clothes were spoiled. But by perseverance he at last got the better of his awkwardness, and acquired the art of experimenting with ease and elegance.

"In 1767 he became one of the head tutors of Trinity College; and in October, 1771, he was appointed Regius Professor of Divinity, with the Rectory of Somersham, in Huntingdonshire, annexed. In 1774, he was presented to a prebend in the church of Ely; and in January, 1780, he succeeded Dr. Charles Plumtre in the archdeaconry of that diocese.

"He had been tutor to the late Duke of Rutland when his Grace resided at Cambridge. In 1782, the Duke presented him to the valuable Rectory of Knaptoft, Leicestershire; and, in the same year, through the recommendation of the same nobleman, he was advanced to the Bishopric of Llandaff. In consequence of the smallness of the revenues of this Bishoprick, Dr. Watson was allowed to hold with it the Archdeaconry of Ely, his Rectory in Leicestershire, the Divinity Professorship, and the Rectory of Somersham. This bishopric was the last of his preferments. Though it is the poorest in the gift of the crown, and though Dr. Watson was, without doubt, one of the greatest ornaments of the bench of Bishops, and though his scientific and theological knowledge, the urbanity of his manners, and his uniform zeal for religion, would have made him fill with honour the first place in the English church, yet there were some circumstances which effectually prevented his promotion.

"He had early associated himself with the political party known in Great Britain by the name of Whigs. During the King's illness in 1789, when the Regency Bill was before Parliament, he took an active part in advocating the right of the Prince of Wales to be appointed Regent without limitation. During the American war, he had been equally hostile to the ministerial party at that time in power, and argued the cause of the Americans with zeal and ability. At the commencement of the war with revolutionary France he was equally an enemy to hostilities; though long before the termination of that memorable struggle he had become sensible of its necessity, and urged its continuance and vigorous pursuit with much earnestness and eloquence. Thus his political sentiments, during almost the whole of his life, were at variance with those in whom lay the virtual distribution of promotions in the church. It is not surprising, therefore, that he was overlooked; though it would have redounded highly to the credit of Mr. Pitt, and his companions in the administration, if they had disregarded all political differences, and contributed to the promotion of one of the greatest ornaments of the church of England; but such want of magnanimity is rather an object of regret than of surprise.

"Dr. Watson

"Dr. Watson distinguished himself as a theological, political, and scientific writer. Of his theological writings, the most important, perhaps, are his Apology for Christianity, and his Apology for the Bible. The first was published in 1776, as an answer to Gibbon's celebrated 15th chapter on the Causes of the Growth of Christianity. Of all the answers to Gibbon, this displayed the greatest urbanity and politeness, without any deficiency either of argument or spirit. The second was published in 1796, in answer to Thomas Paine's Age of Reason, which, at that time, was disseminated with particular industry through the British empire; and, being written in that peculiar style of which the author was such a consummate master, was particularly calculated to make an impression upon the common people. Indeed, the impression which it did make was wonderful, and almost instantaneous. The Apology for the Bible was written to counteract the effects of this insidious book in the only quarter where they could be dangerous; for the ignorance of Paine, and the absurdity of his arguments, were so obvious, that the book could make an impression only on those who were altogether ignorant of the subject, and had never thought of the evidences upon which their religion rested. Dr. Watson's answer, accordingly, was adapted to the understandings of the common people, and was a masterpiece, whether we consider the skill with which he overturned the insidious arguments of his antagonist, or the ability with which he counteracted the baneful effects of the principles which it was the object of the revolutionists to inculcate: for the avowed intention of these wild enthusiasts was to destroy the morality and religion of the common people altogether, and thus render them capable of going every length that might suit the objects of the demagogues of the day.

"In 1785, Dr. Watson published a Collection of Theological Tracts selected from various Authors for the Use of the younger Students in the University, in six octavo volumes. This publication deserves high praise for the judgment with which the tracts were selected. They form a kind of theological library, which cannot but be very valuable to the theological student.

"It will not be expected that I should notice here the different sermons which Dr. Watson published during the course of his long life. Indeed I do not consider myself as at all qualified for such a task, having never seen several of them at all, and only glanced over the rest in a cursory manner.

"The political publications of Bishop Watson are still less proper for discussion in a work like the present, which professes to be totally devoted to physical science. I may notice merely a few of his most prominent opinions. In 1783, immediately after his promotion to the Bishopric of Llandaff, he published a letter to Archbishop Cornwallis on the church revenues, recommending a new disposition, by which the bishoprics should be rendered equal to each other in value, and the smaller livings be so far increased in income, by a proportionate deduction from the richer endowments, as to render them a decent competency.—From the commencement commencement of the discussion respecting the slave-trade he was always a strenuous advocate for its abolition. He also exerted himself strongly in endeavours to repeal the Corporation and Test Acts.—His scheme for the abolition of the national debt, by every individual giving up a certain portion of his property, indicated less refined notions respecting political economy than might have been expected from a writer possessed of so much general knowledge upon so many subjects, and so conversant with the best writers of his time.

"His chemical writings will claim a greater share of our attention. Indeed, it was to give an account of them that the present article was drawn up. In 1769 he was elected a Fellow of the Royal Society; and five papers by him were published in the subsequent volumes of the Philosophical Transactions. These papers were as follows:—

"I. *Experiments and Observations on various Phenomena attending the Solution of the Salts.* (Phil. Trans. 1770, p. 325.)

"This is a very elegant paper, and may serve as a model for the method of drawing up experimental investigations. It first made chemists acquainted with four sets of facts of considerable importance. 1. That, when salts are dissolved in water, they are not merely received into the pores of that liquid, but enter into chemical union with its particles. 2. The specific gravity of different salts. His mode of ascertaining that specific gravity was very simple, and yet susceptible of considerable accuracy. He took a globular glass vessel with a long narrow cylindrical neck. The neck was exactly graduated; so that the proportion which it bore to the capacity of the whole vessel was accurately known. The vessel being filled with distilled water up to a given mark in the neck, a certain weight of the salt whose specific gravity was to be determined was thrown into it. The water immediately rose in the neck of the vessel. From this rise it was easy to infer the specific gravity of the salt."

Of the various kinds he has given a table, the accuracy of which has never been disputed.

"The solution of all salts in water is greater than the mean of the specific gravity of the salt and of the water. Hence a condensation obviously takes place during the solution; and, of course, as the solution goes on, the water is constantly sinking in the neck of the mattrass, though it never sinks so low as the mark at which it stood before the addition of the salt. We may infer from this fact that Dr. Watson's method for determining the specific gravity of salts answers worst for those that dissolve most rapidly in water."

A table was given exhibiting the specific gravity of water at 41, saturated with different salts; and another of the specific gravity of water holding in solution $\frac{1}{12}$th of its weight of different salts. The paper contains also the well-known table of the specific gravity vity of water impregnated with thirty-seven different proportions of the common salt of commerce, varying from $\frac{1}{3}$ of the weight of the water to $\frac{1}{1024}$.

"On Feb. 12, 1771, about an hour after sun-set, the thermometer at Cambridge stood at 6°. This cold seems to have come on rapidly, for the Cam was not frozen. Dr. Watson relates the state in which he found several saturated solutions of salts in his laboratory. But, as he neglected to specify the temperature of these solutions when examined, the information conveyed in this paper is much less valuable than it otherwise would have been."

"In the beginning of July, 1772, he exposed a thermometer to the direct rays of the sun. It rose to 108°. On blackening the bulb by means of China ink, the thermometer rose to 118°. This experiment was carried much further by De Saussure, and the late Professor Robison, of Edinburgh. Saussure, by surrounding the bulb with charred cork, and exposing it to the sun's rays, made it rise to 221° at Geneva. Professor Robison, at Edinburgh, raised a thermometer by similar means to 237°."

Some experiments have been since made to show the difference between a black surface highly varnished, and one equally black without varnish. This forms a new discovery concerning radiant and absorbed heat, which, at that time, was not sufficiently understood when applied to dark colours.

Dr. Watson's two remaining papers in the Transactions are "Chemical Experiments on Lead Ore," and "Observations on the Sulphur Wells at Harrowgate." Of these we need only observe, that, though the subsequent improvements in chemistry have taught us much more than the papers contain, yet they are correct as far as he carried his researches.

"In the year 1781, Dr. Watson published his Chemical Essays, in three 8vo. volumes. This work was highly popular at the time of its appearance, and contributed very materially to produce that taste for chemical science which at present so generally pervades Great Britain. These essays are, beyond dispute, the most elegant work on chemistry which has appeared, either in this country or the continent of Europe. Though the science has undergone two complete revolutions since 1781, and though the progress which every branch of it has made since that period is quite enormous, yet these essays have not yet lost their interest, and will always retain a considerable portion of value, because they contain a great collection of historical facts not to be found any where else. The essays on gunpowder, on the saltness and temperature of the sea, on the quantity of water evaporated from the surface of the earth in hot weather, on the smelting of lead ore, on silver extracted from lead, and on red and white lead, may be still perused with interest by the chemists of the present day, and deserve to be studied as models of elegant memoirs on scientific subjects. It would far exceed the limits to which I must confine myself here, were I to attempt an analysis of the contents of these volumes.

 But

But I consider the task as quite unnecessary, as they must be familiar to every chemist of taste in the British empire.

"In the year 1786 he published a fourth volume of Chemical Essays, which I consider as much more valuable than the preceding ones. It contains a great stock of very valuable information respecting the progress of various chemical manufactures in Great Britain, chiefly concerning the smelting of some of the metals, and forming some of the most important alloys. His account of blende, zinc, brass, gun-metal, tinning copper and iron, and gilding in *or moulu*, are highly interesting. To this volume he soon after added a fifth, containing his essays which had been published in the Philosophical Transactions, and one or two others of inferior consequence.

"It is very much to be regretted that Dr. Watson did not continue to prosecute the science of chemistry, which he had begun to cultivate with so much ardour, which his erudition and sagacity fitted him so well to have improved, and his elegant style so well qualified him to explain."—*Gent. Mag. and Annals of Philosophy.*

Dr. Watson died in July, 1816, having nearly completed his 79th year.

---

The following account of Dr. Rush's death is extracted from Mr. Pettigrew's Memoirs of Dr. Lettsom. Of this work we shall give a further account in our next.

"Dr. Mease to Dr. Lettsom.

"*Philadelphia, Dec.* 21, 1815.

"Dear Sir,—I had lately the pleasure to receive your 'Recollections of the late Dr. Rush,' for which I thank you. I was much concerned, however, to find, that you had given currency to the incorrect statement propagated after his death, 'that he had mistaken his disease for the pleurisy, and was bled freely, which was thought to have occasioned his death.' Your correspondent was unfortunately misled by common report, which is too often incorrect; and in the present instance this incorrectness is to be particularly regretted, because it favors the diffusion of error, and implicates the medical judgment of a man, who was more extensively consulted by his countrymen than any other physician that ever lived in the United States; and it gratifies the little and mean spirits among us,* who exulted in the report of his having fallen a victim to his attachment to the depleting system, and who will be glad to find that the report has been circulated in Europe. Dr. Rush was not afflicted with '*typhus or spotted fever*,' but a true pleurisy; and the blood, so far from being '*freely*' taken, amounted only to ten ounces in quantity: more was not taken away (except locally), although the pain in his side, after having been relieved by the operation, returned with severity; and the disease ended as

* And *among* us also. See our former remarks on this subject. —Edit.

inflammatory inflammatory affections of the lungs often do in such habits as that of Dr. Rush. The case was strictly as follows:

"Dr. Rush, in the early part of his life, had been subject to a cough, which he kept under by occasional small bleedings, great temperance in diet, and by a careful accommodation of his dress to our inconstant climate. He had been attacked by a cough several months previously to his last illness, and in consequence of it, he had abridged his customary proportion of animal food (in the use of which he was at all times very moderate), and left off entirely the use of wine.

"The effects of these retrenchments alone would have been felt by frames more vigorous than that of Dr. Rush; but in his case, and at his time of life, they could not fail greatly to diminish the muscular power, and increase the excitability of his system, to the causes that produced the fatal disease. Other causes co-operated. During the time alluded to, he was engaged in extensive practice, had performed his tour of duty at the Pennsylvania Hospital, and at the close of the session in March had given two lectures daily, of an hour each: he also assisted in the examination of a large class of candidates for medical degrees in the university of Pennsylvania, twice a day, and at night he either was engaged in study, or in answering the numerous letters of applicants for medical advice from every part of the continent. Thus, by such unremitted corporeal and mental exertion he wasted the powers of life, and predisposed his system to the operation of the variable atmosphere that caused the affection of his lungs.

"He was attacked by his last illness on the night of the 14th April, 1813. I had been absent from the city, and on my return called to see him in the evening. I found him alone with a lecture before him, and a pen in his hand. Having before hinted to him that he ought to relax in his studies, I said, 'What, doctor, always at your studies?' he replied, 'Yes, doctor, I am revising a lecture, for I feel every day more and more like a dying man.' Alas! how prophetic his words! Upon my observing that I hoped he did not feel indisposed, he replied, 'No; but at my age I deem life particularly precarious, and I am, moreover, anxious to leave my manuscripts as perfect as possible for the benefit of my son. We conversed for an hour or more upon various medical subjects, and I heard him read an affectionate letter to a relation in a distant state; who had asked his opinion upon an important occasion. A person having called for a letter of advice, I retired to another room, where I remained near an hour with his family. Upon returning to him, I found him sitting with his feet close to the fire, and, after a moment's stay, I wished him good night. Mrs. Rush came in as I went out; and I subjoin her own statement of the progress of his attack, and the remedies used. This statement was drawn up at my request, that there might be no doubt as to the accuracy of every particular on the distressing subject:

"'At nine o'clock in the evening of Wednesday the 14th April 1813, Dr. Rush, after having been as well as usual through the day, complained complained of chilliness, and general indisposition, and said he would go to bed. While his room was preparing, and a fire making, he became so cold, that he called for some brandy and drank it; he then went to his room, bathed his feet in warm water, got into a warm bed, and took some hot drink: a fever soon came on, attended with great pain in his limbs and in his side; he passed a restless night; but after day-light a perspiration came on, and all the pains were relieved except that in his side, which became more acute. He sent for a bleeder, and had ten ounces of blood taken from his arm, with evident relief. At ten o'clock, Dr. Dorsey saw him, heard what had been done, and approved of the treatment, observed that his pulse was calm, but rather weak, and advised him to drink plentifully of wine whey, which was immediately given to him. He remained the rest of the day, and on Friday, with but little apparent disease, though never quite free from fever, and always complaining when he tried to take a long breath. On the morning of Saturday he awoke with an acute pain in his side, and desired that the bleeder might be sent for: to this I objected on account of the weak state of his pulse. I proposed sending for Dr. Dorsey, but Dr. Rush would not consent to his being disturbed. He reminded me of his having had a cough all the winter, and said, ' this disease is taking hold of my lungs, and I shall go off in a consumption.'

" ' At eight o'clock, Dr. Dorsey saw him, and, upon feeling his pulse, objected to his losing any more blood, and called in Dr. Physick, who agreed in the opinion that bleeding was improper. The pain in his side, however, continuing, and his breathing becoming more difficult, Dr. Physick consented to his losing three ounces of blood from his side, by cupping. This operation relieved him, so that he fell into a refreshing sleep, and towards the evening of Saturday his fever went off, and he passed a comfortable night; and on Sunday morning seemed free from disease. When Dr. Physick saw him, he told me that Dr. Rush was doing well, that nothing now appeared necessary but to give him as much nourishment as he could take. He drank porter and water, and conversed with strength and sprightliness, believing that he was getting well, until about four o'clock in the afternoon, when his fever came on again, but in a moderate degree. At five o'clock, Dr. Physick and Dr. Dorsey visited him, and found him not so well as in the morning, but not appearing to apprehend what so soon followed; for at that time nothing was ordered different from the morning. At nine o'clock, they again visited him, when they found him so low as to apprehend a fatal termination of his disease. Stimulants of the strongest kind were then administered: you, my friend, know with how little effect!'

" I was constantly with Dr. Rush all the next day, and witnessed the progress of that debility which deprived me of my friend, the medical republic of its ornament, and our country of one of its best men, and the early, steady, and zealous supporter of our independence.

"It

"It was the greatest absurdity to suppose that any man, acquainted in the least with medicine, could mistake pleurisy for typhus, the symptoms of which diseases are so different. The loss of the blood taken away from Dr. Rush would have been prescribed in his case by any medical man in Philadelphia; and I fancy that the remedy would have been sanctioned by the greater part of European physicians, strong as are their prejudices against the operation; for, although it certainly weakened him, yet had it not been ordered, it is highly probable that the excessive pain in his side, which it is evident was firmly fixed a few hours after his first indisposition, from its not being removed as the other pains were by the perspiration that came on the first night of his illness, nor by the bleeding,—and the great difficulty of breathing, which showed pulmonary congestion,—would have exhausted him even before the period at which the disease finished its course.

"It is with difficulty that I can refrain from dwelling on the character of Dr. Rush, his eminent talents and virtues; and from stating the strong claims which his memory has from his country to veneration, from his zealous exertions as a patriot of our truly glorious revolution, and in promoting the establishment of our excellent federal constitution, notwithstanding the shafts of calumny directed against him on both occasions; and how much Philadelphia owes him for the benevolent, civil, patriotic, and religious institutions which he either originated or powerfully supported; and for assisting in raising the medical character of our university and city to the proud station it holds in the world; from the humane exertion of his medical talents in the service of the distressed sick, and the influence of his example upon others. These traits I leave to his biographer, whoever he may be. My intention in addressing this communication to you, was only to correct what I deemed an important error as to the cause of his lamented death; and having done so,—I hope to your satisfaction,—I beg you will accept of my sincere wishes for your health and happiness.

"James Mease."

*On the Evolution of Heat during the Coagulation of the Blood.*

We know not whether the Dr. Gordon mentioned in the following ingenious paper is the person who has been accused of undervaluing Hunter and Abernethy in the Edinburgh Review, and of abusing Gall and Spurzheim in the same publication. If so, he certainly affords one of the most striking instances of Mr. Hunter's just observation, that few people are aware of all the results of experiments which are at all complicated.

It is well known that Dr. Gordon found, as he conceived, several degrees of heat evolved by the coagulation of blood. This was contrary to Mr. Hunter's observation, which Dr. G. imputes to Mr. Hunter's not moving the bulb of the thermometer from the fluid to the more solid part of the blood, so as to ascertain the difference. We shall see Dr. Davy's remark on this supposed

 oversight oversight in one whose discoveries, and the inferences he drew from them, stand, in most instances, uncontradicted.

*On the Heat evolved during the Coagulation of Blood.* By J. DAVY, M.D. F.R.S.—From the Journal of Arts and Sciences, p. 246.

"WHETHER any heat is evolved during the coagulation of blood, is a question which has received opposite answers from different enquirers.

"My friend, Dr. Gordon, is decidedly of opinion, that the phenomenon alluded to, is attended with a considerable elevation of temperature, amounting even to several degrees.

"This opinion of his I ventured to controvert, in my Inaugural Dissertation, published about two years ago. And Dr. Gordon did me the honour of replying to my remarks, in a paper published about eighteen months since, in Dr. Thomson's Annals, in which he maintains his former doctrine.

"At present it is not my object to criticise his essay, but to offer some additional facts relating to the subject under dispute, which I had an opportunity of collecting on my voyage to this place, in my way to India. My experiments were made on the blood of the turtle and shark, which last is extremely well adapted to the purpose, as its temperature approaches nearly to that of the atmosphere; and on the blood also of sheep.

"On the 15th of March, when our ship was in latitude 4° 9′ N. and longitude 19° 15′ W. by chronometer, at sun-set, a large shark was taken, by means of a harpoon. As soon as it was brought on deck, whilst it was still alive, it was cut in two. The blood flowing in the great dorsal vein was 82°;* the surrounding thick muscles were 82,5°; the water of the sea was 80,5°; and the air 79°. Some of the blood was collected in a glass. In about two minutes it had firmly coagulated. During the whole time I watched the thermometer immersed in it. The mercury sunk from 81,5° to 81°, and did not rise at the instant that the coagulation commenced, nor did it remain stationary whilst the coagulation was going on, but continued gradually sinking.

"The day following, another shark was taken. The same experiment was made with the blood, and a similar result was obtained.

"On the 23d of March, when we were in latitude 2° 29′ S. and in longitude 24° 30′ W. a large turtle was killed, which had been caught about three weeks before, at the island of Ascension. The air at the time was 79°. The blood of the turtle flowing from the carotids was 91°. When collected in a glass it was 88,5°. The thermometer, placed in the midst of it, immediately began to fall,

"* This result is an additional evidence that venous blood is of a lower temperature than arterial; a circumstance which I have endeavoured to prove by numerous observations contained in the Thesis already alluded to."

and and continued falling gradually, without any sensible interruption, whilst the blood was coagulating.

"Since I have been in Cape Town, I have repeated my experiments on the blood of sheep. To enter into any details of them would be superfluous, since the results they afforded agreed perfectly with those already described. The air being about 60°, and the blood when drawn about 100°, it continued cooling whilst its coagulation was going on, so that, when the coagulum had formed, its temperature was diminished, in between two and three minutes, about one degree and a half.

"From these experiments, the obvious, and, it appears to me, the unavoidable conclusion is, that which I had before adopted in my Thesis, and which had been first drawn by Mr. Hunter, viz. 'That during the coagulation of blood, there is no sensible evolution of heat.'

"It now only remains for me to reconcile the fact, (if I may venture to call it by that name,) with the well-established principle, that change of temperature is the necessary consequence of the change of form of bodies in general; and to suggest a reason for the difference of Dr. Gordon's and my own results.

"To accomplish the first, there is little difficulty; and the explanation which I proposed in my Thesis I shall again offer, as it seems to me quite adequate to the purpose.

"Since, during the coagulation of blood, a part of it passes from the liquid into the solid state, there should be, according to theory, some increase of temperature. But, since this liquid part, the fibrine, which becomes solid, is so small as to amount only to about $\frac{1}{50}$ of the whole quantity by weight; and since the coagulation is not an instantaneous, but a slow and gradual effect, it appears to me as necessarily to follow, that the heat produced must be too slight to affect sensibly the thermometer. This granted, which may be proved to demonstration, the anomaly vanishes,—this fact no longer opposes the general principle.

"The difference between the results of Dr. Gordon's experiments and my own, has arisen, perhaps, from the different modes in which our experiments were made. Dr. Gordon, I think, kept the bulb of his thermometer near the bottom of the vessel containing the blood, and when this fluid began to coagulate towards the surface, he drew the instrument up. On the contrary, in all the experiments I have detailed in this paper, the thermometer was not allowed to remain stationary; it was gently moved from one part to another, so that the whole might be kept of the same temperature till the coagulation commenced: for, when the blood is viscid, and the vessel deep, the surface on the part last drawn is warmer than that below, and when shallow, the bottom is warmest, as I have frequently observed in my experiments, and as I have remarked in my Thesis, as a source of inaccurate observation.

"I could wish to enter more into detail upon the subject; but, at this distance from Europe, and consequently from every scientific journal and scientific work, it is out of my power. To Dr.

Gordon's well-known candour and liberality of mind I must trust for pardon, for any oversight I may have committed in the consideration of his paper. I am, however, confident, that, if Dr. Gordon will repeat the experiments I have described, he will obtain the same results, and be satisfied with Mr. Hunter's original conclusion.

"*Cape of Good Hope;*
*May* 24*th*, 1816."

For Dr. Gordon's paper, see our Journal, vol. xxxii. page 223; and for Mr. Davy's former paper, see vol. xxxiii. p. 113.

Highly as we respect the physiological powers of Mr. Hunter, and the chemical accuracy of Dr. Davy, we cannot help suggesting, purely for information sake, whether there is not yet something undecided in the question. Mr. Hunter's experiments, and some of Dr. Davy's, were made on cold-blooded animals. In these we might, indeed, have expected the common chemical effects of the evolution of heat by consolidation; but not the generation of heat from action during animal life; because these animals have not a standard heat. Dr. Davy's last experiments were made on sheep, who possess that property. From a perusal of his very accurate and frequently-repeated remarks, we venture to suggest whether the experiment might not be best made in *a deep* vessel, graduated to 100°, and the thermometer raised, not to the surface, but to the lowest part of the coagulum when sufficiently formed?

We have, also, a remark to make on Dr. Gordon's paper. His first experiment was on blood taken from the femoral artery of a dog. The blood began to coagulate *one minute* after it had been received into the vessel. This was earlier than commonly happens, and speaks a greater degree of action, consequently a probability of more heat than in the ordinary course of things. In this instance the thermometer was raised six degrees of Fahrenheit. When the experiment was made on human venal blood, it was always during inflammation, and the thermometer was raised twelve degrees Fahr. Here, then, was increased action, and such an increased action as is always attended with increased heat in the living body: Whether this had any influence on the different results, we pretend not to say. On turning to Mr. Hunter's Treatise, p. 27 and 28, we see that his first experiment was made on human blood, with the caution of raising the vessel to a proper temperature, as we have suggested above; yet even then the blood, instead of becoming warmer, cooled rather faster than other blood placed in the same temperature, but not in the act of coagulating.

---

### *Instructions to the Apprentices of Surgeons.*

It has long been our intention to answer, in a systematic form, the very laudable enquiries of Juvenis concerning the best mode of improving his time during his apprenticeship. Our only objection has been, that the far most considerable part of our readers might might feel little interest in such a subject. Yet many of these may have sons or young people whom they would be glad to see engaged as Juvenis wishes to be. To others we can only say, that we shall be thankful for their animadversions on our own remarks, or for their original observations.

Introductory to the subject, we have selected a few passages from Mr. Stevenson's* Oration before the Medical Society of London. We hope that gentleman will not feel offended at our presenting in writing what he apologized for offering orally so soon after the unexpected demise of Mr. Royston. Perhaps, however, the very haste with which the oration was written may be to its advantage, as it is not uncommon to spoil a good composition by too many attempts at refinement. Our limits will permit only such passages as are particularly to our purpose, by showing the dignity of our profession, the manner in which it has been studied at different periods, and, most of all, the advantages enjoyed by the present generation.

For this reason, we take no notice of the very eloquent apostrophe concerning the human fabric, its liability to disease or accident, and the consequent necessary introduction of medicine in some form at the earliest period of mankind; nor of the rude state of the art under the Babylonians and Egyptians. In reverting to its improvement by the Greeks and Romans, the orator descanted with equal learning and eloquence on the writings left us by Hippocrates, Galen, Celsus, and Aretæus. Of these he remarked, that, having few remedies, and a very imperfect knowledge of human, still less of morbid, anatomy, their practice must have been feeble, and, for the most part, the offspring of mere experience. Yet these very circumstances obliged them to attend more closely to the form of disease, which they could often trace throughout, without confounding its true symptoms with the changes induced by our more powerful remedies. For this purpose, their writings are worth the care with which they have been preserved, and themselves to a portion of those divine honours which were bestowed on the first family of physicians. If the employment is now less dignified, it is more important in proportion as it is better understood.

It was well observed, that, "by the proper exercise of this godlike art, we possess the enviable power of doing good to our fellow-creatures, in a way the most necessary to their wants, and most interesting to their feelings. By this we are enabled to confer that which sick kings, if cut off from the source of relief, would not hesitate to purchase at the price of their crowns;—that which the most exhaustless wealth alone cannot command, nor state nor rank bestow. We shall be able, by the diligent attainment and exercise of medical knowledge, to alleviate and remove disease, the most distressing and the most insupportable of human afflictions; and to substitute the blessing of health, 'which he who

* Oculist and Aurist to several of the Royal Family.

possesses possesses has little more to wish for, and he who is so unhappy as to want wants every thing with it.' Hence, physicians, properly qualified, and duly fulfilling the duties of their important vocation, have, in most civilized countries, been held in the highest veneration and respect, and, in some foreign states, have been advanced to lofty civic honours."

The profession of physic, Mr. S. remarked, although not possessing so many allurements as those of divinity and law, is by no means barren of attractions. It opens the way to reputation and wealth, and raises the successful practitioner to a level, in the intercourse of common life, with the highest classes of society. It must be acknowledged, however, that the exercise of the medical profession is not always attended with the most pleasing intercourse. It views human nature in the most unfavourable and too often fretful moments, when it is almost impossible to please, or to avoid even offence by its best services; so much foundation is there for Dr. Johnson's very striking picture of it, viz. "that it is a melancholy attendance on misery, a mean submission to peevishness, and a continual interruption to pleasure."

The requisite qualifications to enable a physician to practice the arduous duties of his vocation with advantage were next detailed. The communication of medical knowledge through the medium of the discussions of the Society were ardently recommended; and an eloquent tribute paid to the memory of the deceased President, Dr. Lettsom.

After remarking on a few of the errors that have at different times prevailed from the introduction of ill-founded theories, Mr. S. justly observed, that "it is hard to say what is the prevailing theory of disease. Many morbid phenomena have been referred to the mysterious agency of electricity and galvanism." "Our attention is, at present," said our author, "more particularly directed to the functions of the digestive organs; and, of these, the liver has had the imputation of giving birth, like Pandora's box, to almost innumerable descriptions of evils. The bile, in the character of excess or defect in quantity, or vitiation in quality, is reproached with being the prolific parent of a dreadful catalogue of nervous, dyspeptic, arthritic, and various other complaints, of the most discordant nature and protæum form." The due and healthy action of this viscus, however, the orator admitted, is of the greatest importance in the œconomy.

Our limits will not admit of our following the learned orator through his ingenious view of this subject: we must, therefore, hasten to conclude, by noticing a few remarks made by Mr. S. respecting Amaurosis. "This disease is universally admitted to consist in an impaired or total loss of sensibility of the nerve appropriated to the sense of vision, the tunics and humour of the organ retaining their natural transparency. Whatever causes are capable of deranging the functions of the optic nerve, by acting directly upon its origin in the brain, or its expanded termination in the retina, or, lastly, by its sympathy with some remote viscus, as as the stomach, may (said Mr. S.) become the exciting cause of this complaint. Now, as these are various, so must the modes of treatment be equally diversified. Hence it follows, that, to attempt the cure in all instances by the same means, as is too commonly done, by stimulants and nervines, on the supposition that the disease arises from a weakness or torpor of the nerve, must be no less irrational than unavailing.

During the last few months, Mr. S. had met with several cases of this disease, characterized by so complete a loss of sight, that the patient was able to distinguish only the outlines of objects, or luminous rays, from total darkness. The pupil was fully and immoveably dilated, without any appearance of fulness of the vessels of the conjunctiva, or more than a sensation of very slight tensive uneasiness in the globe of the eye. The disease commenced, in all the instances, very suddenly, chiefly in the night; and was not preceded by any of the usual precursory symptoms. The patients were of both sexes, of various ages and stations in life. In some subjects one, in others both eyes were affected, without any preceding or accompanying indisposition. After very careful and minute enquiry, Mr. S. succeeded in tracing the origin of the attack to exposure of the eye to the partial application of cold air, when the body was preternaturally heated.

With regard to the pathology of the disease, time would not allow him to enter into particulars, which, however, we hope he will shortly be induced to lay before the public. He seemed disposed to suspect, that the blindness in these cases arose from the sudden repulsion of blood from the superficial to the deep-seated vessels of the eye, in which, perhaps, there might previously exist a *partial* plethora. "The defect, therefore, of vision in these instances," Mr. S. suggests, "is probably the consequence, not of an indefinable and inexplicable torpor, but of a direct compression of the fibrous substance of the optic nerve, by the sudden enlargement of its central artery, or of its medullary expansion in the retina, by the blood-vessels so abundantly distributed through its pulpy texture becoming, from the above cause, incidentally and preternaturally over distended." Without contending for the accuracy of the theory suggested, Mr. S. had the satisfaction to add, what is of much greater importance, that, by means of active general and topical depletion, each of his patients, in the several cases to which he alluded, obtained the speedy and complete restoration of sight.

The Oration was very numerously and respectably attended; and there is reason to regret, that the learned Orator could not be prevailed upon to print it, as it is a production equally creditable to the Society and its author.

